# Giant Coronary Artery Aneurysm Arising From a Septal Perforator: Diagnosis and Percutaneous Management

**DOI:** 10.1016/j.jaccas.2026.109098

**Published:** 2026-07-01

**Authors:** Mariam Dvalishvili, Ahmed Ghoneem, Constantine Marcu, Paul Mahoney

**Affiliations:** a Department of Cardiovascular Sciences, East Carolina University, Greenville, North Carolina, USA; b Department of Cardiovascular Medicine, University of Pittsburgh Medical Center (Harrisburg), Harrisburg, Pennsylvania, USA

**Keywords:** coiling, giant coronary artery aneurysm, septal branch aneurysm

## Abstract

**Objective:**

Coronary artery aneurysms (CAAs) are rare, and management is often uncertain. We present a case of a massive CAA arising from a large septal perforator branch of the left anterior descending coronary artery that was treated successfully with coiling with complete obliteration of the aneurysmal cavity.

**Key Steps:**

The coiling strategy was 2-fold: intrasaccular coiling to promote thrombosis of the aneurysmal cavity and coil embolization of the feeding septal perforator branch to eliminate residual flow. Angiography confirmed complete occlusion of flow to the aneurysm and the septal branch. Regression in aneurysm size was appreciated on follow-up computed tomography.

**Potential Pitfalls:**

Coiling CAAs may result in incomplete occlusion, coil migration/embolization, conduction disturbances, mechanical injury (vessel dissection or perforation), aneurysm rupture, and ischemia or myocardial infarction. Long-term outcome data are scarce in the literature.

**Take-Home Messages:**

When anatomy permits, transcatheter coiling via a feeding branch can fully obliterate a giant septal CAA and represents a viable alternative to surgical approaches. Multimodality imaging is essential for diagnosis, procedural planning, and postprocedural assessment.

## Visual Summary

**Figure:**
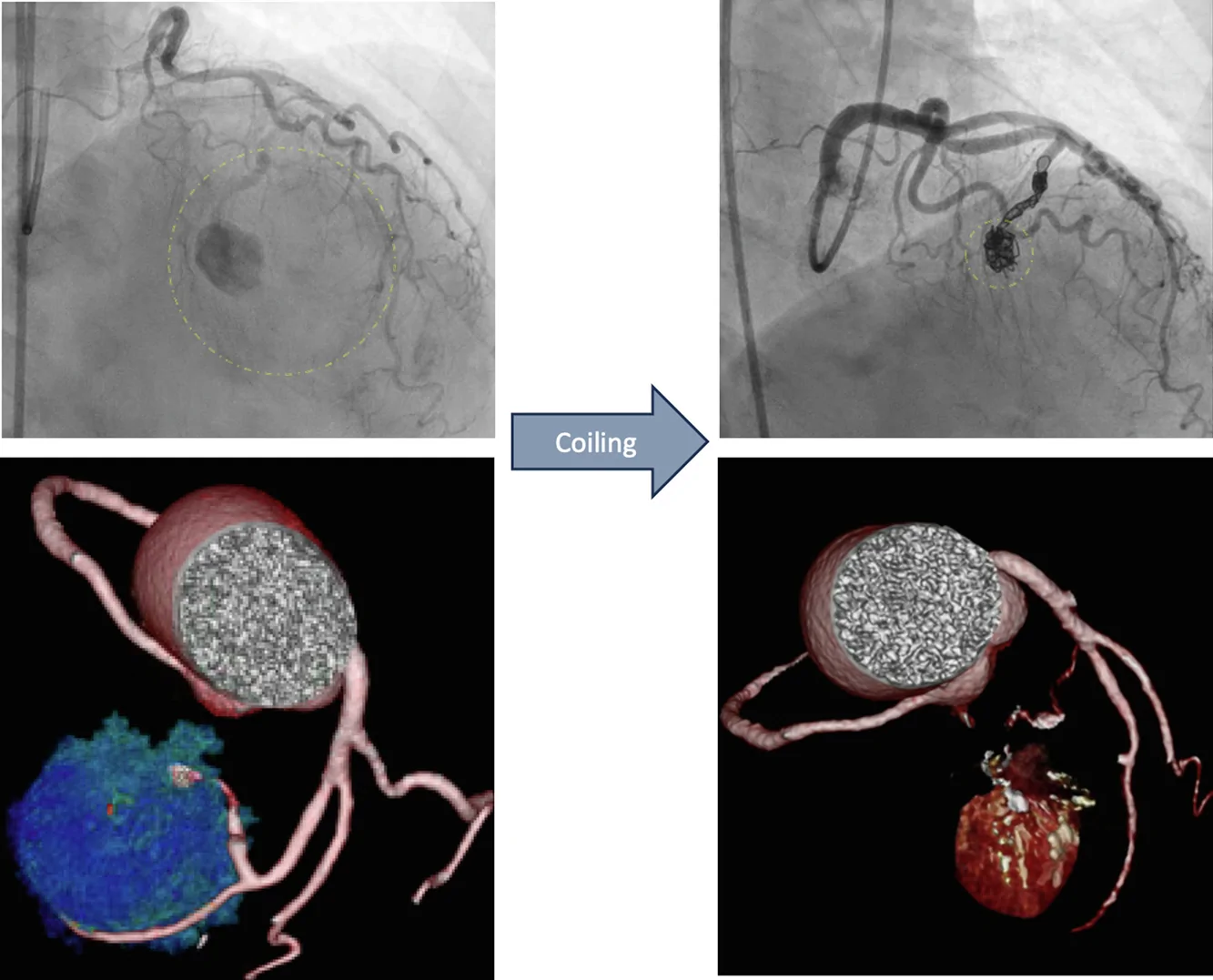

The incidence of coronary artery aneurysms (CAAs) ranges from 0.3% to 5.3%, whereas giant CAAs are even more uncommon.^1^, ^2^, ^3^ Although no single accepted definition exists for a giant CAA, a commonly used threshold is an aneurysm that is 4 times larger than the adjacent normal vessel segment, or ≥ 20 to 50 mm.^3^^,^^4^ CAAs are frequently incidental findings because patients are often asymptomatic.^2^ The clinical implications of giant CAAs remain ambiguous, though there is evidence suggesting association of CAAs with worse long-term outcomes.^5^ The optimal management strategy for CAAs remains challenging in the absence of randomized controlled trials or even large case series reporting outcomes. Intervention is often considered necessary given that complications from a CAA can be serious or life threatening and include rupture, embolization, thrombosis, arrhythmias, compression of adjacent structures, and heart failure.^2^^,^^4^

CAAs are usually associated with epicardial coronary arteries,^2^ consequently, most literature is predominantly focused on these vessels. Prior case reports suggest that septal CAAs may have pressure effects on adjacent structures and potentially lead to conduction abnormalities such as bundle branch blocks.^6^ Furthermore, whereas case reports have described both successful percutaneous coronary intervention and surgery to treat CAAs, no consensus exists on best practices.^2^ In general, patients with smaller CAAs and patients with high surgical risk are usually managed with percutaneous coronary intervention, whereas surgery is considered for patients with larger aneurysms or patients with higher risk of rupture.^7^ More recently, in a contemporary case series, Vink et al,^8^ described giant CCAs arising from major epicardial vessels managed largely surgically or conservatively, with 1 case treated percutaneously.

To date, only a handful of case reports describe septal perforator CAAs, managed either conservatively or surgically;^6^^,^^9^ however, none of these reports described a septal aneurysm of comparable size to the vessel in this report that was managed percutaneously. To our knowledge, this is the first report of a giant CAA arising from a large septal perforator branch that was successfully treated by a catheter-based therapy.

## Case Summary

**Figure 1:**
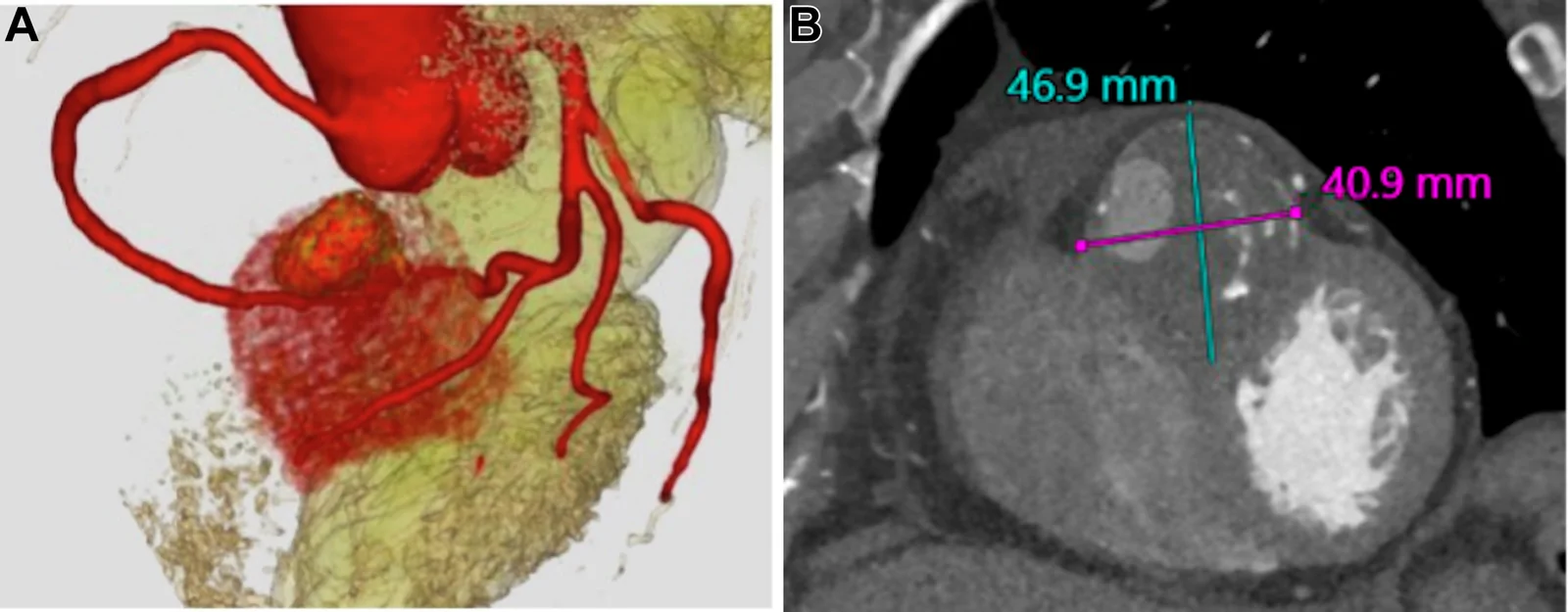
Coronary Computed Tomography Assessment of Coronary Aneurysm Coronary computed tomography showing a three-dimensional rendering (A) and sagittal view (B) of a massive (47 × 41 mm), circular aneurysmal mass, supplied by a large septal branch.

**Figure 2:**
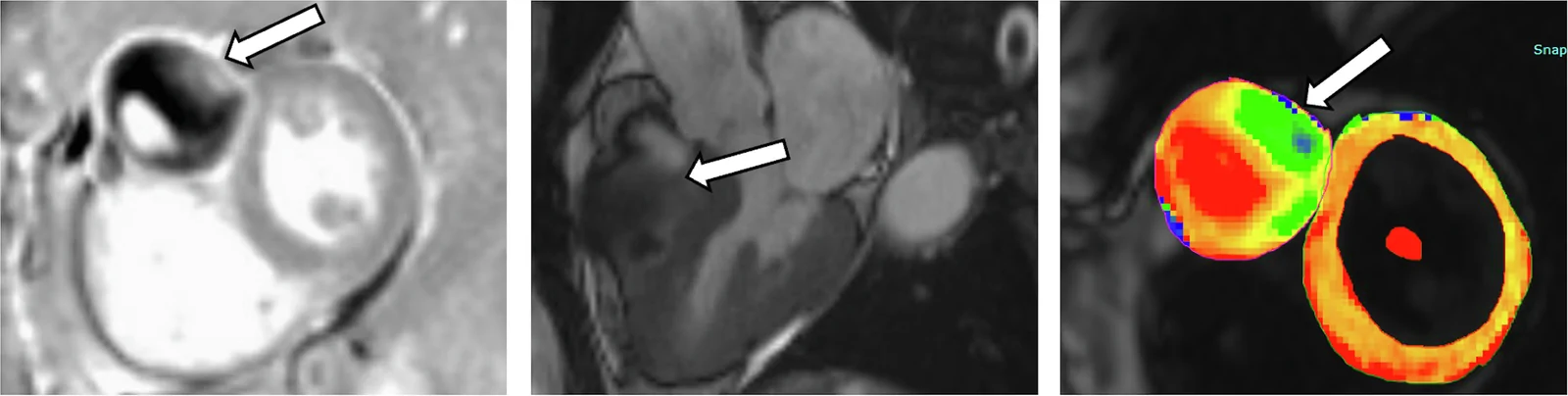
Magnetic Resonance Imaging of Coronary Aneurysm Cardiac magnetic resonance with post-gadolinium contrast inversion recovery and T1 parametric mapping demonstrating a mass in the anterior interventricular groove, containing calcium, thrombus, and blood pool (arrows).

**Figure 3:**
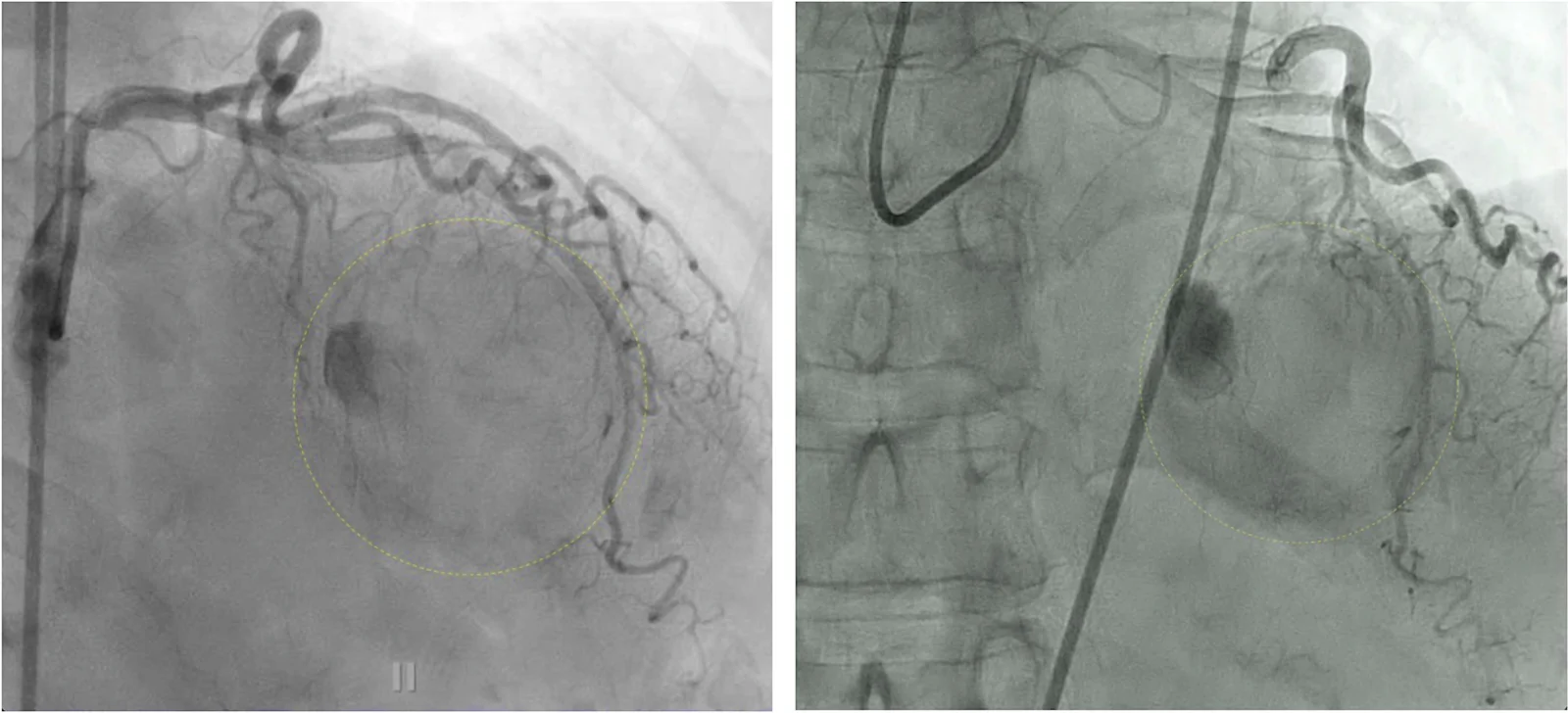
Cine Angiography of Coronary Aneurysm Coronary angiography views demonstrating a large septal perforator branch giving rise to a small contrast-filled chamber surrounded by a large, presumably thrombosed and calcified mass (yellow circles).

A 72-year-old woman with no prior cardiac history was referred to our center after an aneurysm on the anterior aspect of the heart was incidentally identified on computed tomography imaging of her abdomen. We therefore proceeded with a multimodality imaging evaluation of this mass. First, coronary computed tomography angiography (CCTA) was performed, which revealed a 47 × 41 mm round, tapering mass with calcified segments, thrombus, and blood pool in the anterior interventricular groove, communicating with a large septal perforator branch of the left anterior descending coronary artery (LAD) (Figure 1). Next, cardiac magnetic resonance confirmed a well-defined aneurysm within the interventricular septum with similar composition and communication to the septal branch (Figure 2). The study demonstrated normal biventricular function without wall motion abnormalities or late gadolinium enhancement and normal myocardial strain parameters. Transthoracic echocardiography showed mild distortion of the right ventricular anatomy, concerning for mass effect on the right ventricular outflow tract. Finally, invasive coronary angiography showed a large circular aneurysmal mass, supplied by a large septal perforator branch. There was also evidence of competitive flow seen in the LAD from the septal branch feeding the aneurysm (Figure 3, Video 1). The remainder of the coronary tree showed no significant disease.

Although the patient was asymptomatic, the risk of ongoing growth, rupture, and compression of adjacent structures was perceived to be significant by the heart team given the size and intramyocardial position of the aneurysm. Therefore, intervention was considered to be necessary. The septal location and the considerable size of the aneurysm were believed to markedly increase the risk of surgical aneurysmectomy, especially the risk of perforation and loss of cardiac integrity. The team consensus was that an attempt at transcatheter approaches would be reasonable.

## Procedural Planning

Although percutaneous covered stent–based therapies of CAAs have been well documented for aneurysms arising from major epicardial vessels,^2^ we thought the risk of compromising the LAD, which was free of any significant disease, and potential stent-related limitations, including the need for long-term dual antiplatelet therapy and potential risk of residual leak or restenosis, rendered this approach unfavorable. The proximal landing zone in the septal branch and the morphology of the aneurysm provided favorable anatomy for a transcatheter coil-based strategy to induce thrombosis within the aneurysm and to occlude inflow by placing coils within the aneurysm and the septal branch itself. Proximal occlusion of the feeding septal perforator would have been a potential alternative strategy; however, collateral flow may have permitted persistent retrograde filling given collateral flow within the septal branches. Although a focal narrowing was noted, the small caliber and distal location of the vessel limited the feasibility of vascular plug delivery and deployment. In addition, direct sac embolization was favored to promote more immediate thrombosis and complete occlusion, minimizing the risk of residual filling or delayed expansion.

We therefore planned on progressive obliteration of the aneurysmal cavity with coils, while avoiding overmanipulation that might precipitate rupture. The coiling strategy would focus on placing sufficient coils within the aneurysm to promote thrombosis, followed by completely occluding the flow to the aneurysm by placing coils within the septal branch. Follow-up imaging would be planned to confirm depressurization of the aneurysm and obliteration of flow. If coiling was unsuccessful, the surgical approach could still be pursued thereafter.

## Procedural Steps

A 6-F XB 3.5 guide catheter (Cordis) was used to engage the left main coronary artery. A 190-cm BMW wire was advanced initially, but repeatedly selectively engaged a more proximal branch of the septal perforator; a second 300-cm BMW wire was therefore advanced and placed into the aneurysm (Video 2). A FineCross MG microcatheter (Terumo) was advanced over the 300-cm wire into the aneurysm, and the wire was subsequently removed. A total of 4 Azur (Terumo) hydrophilic coils were used. Initially, two 5.0 × 16 mm coils were advanced and deployed within the aneurysm. The microcatheter was then withdrawn slightly, and two 4.0 × 13 mm coils were deployed in the distal and proximal segments of the septal perforator (Video 3). We chose Azur coils given availability and familiarity with this system; however, other hydrophilic coils could have also been suitable. Final fluoroscopic imaging revealed abrupt cessation of flow in the proximal third of the perforator with TIMI flow grade 3 preserved down the LAD. There were no postprocedural conduction disturbances. The patient tolerated the intervention without complications and was discharged the same day on aspirin 81 mg daily.

**Figure 4:**
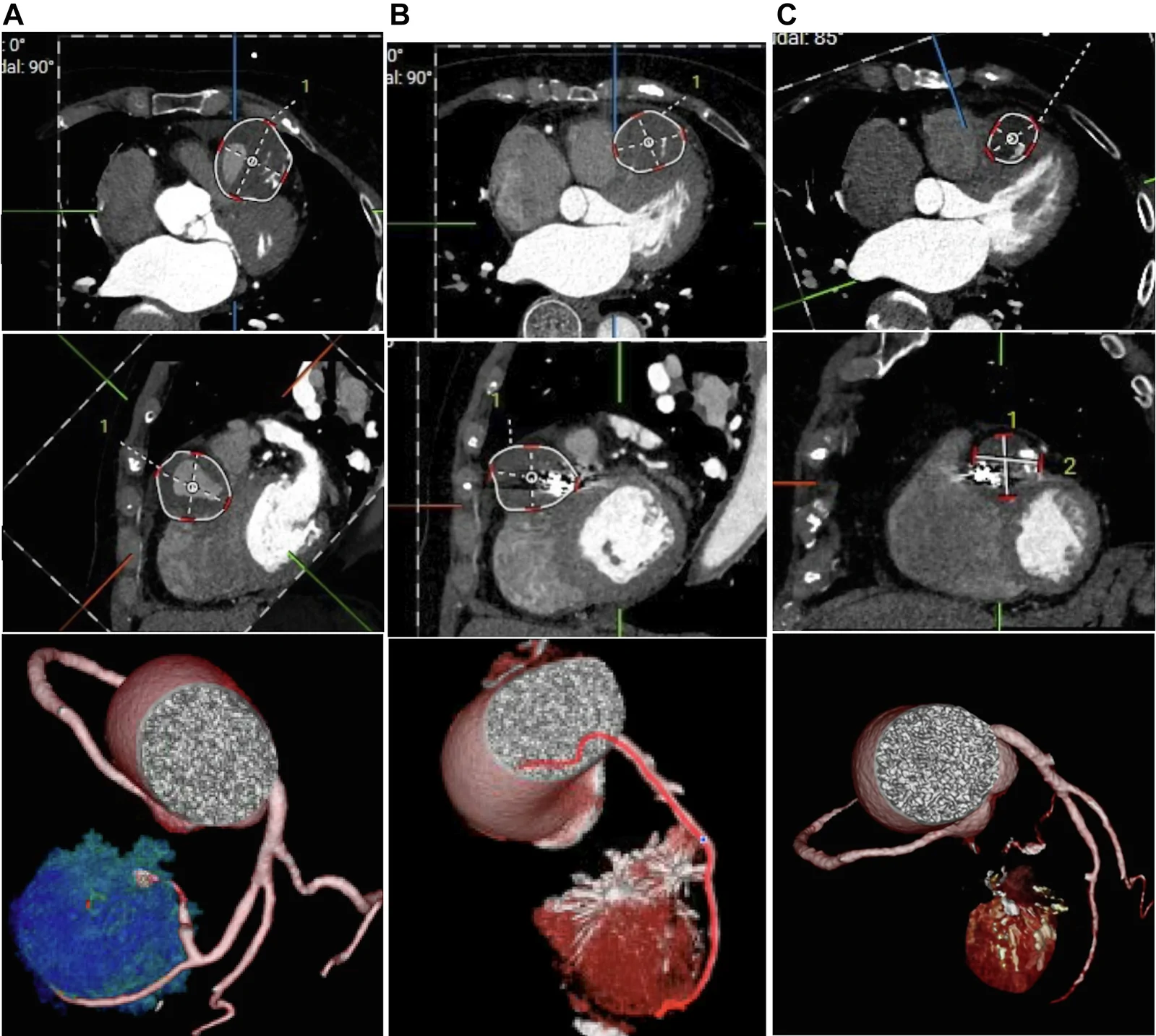
Computed Tomography and Three-Dimensional Reconstruction of Coronary Aneurysm at Baseline and Follow-Up Initial computed tomography imaging and 3-dimensional rendering of the aneurysm at baseline (A) and follow-up imaging at 1 month (B) and 6 months (C). Measurements were obtained at the largest aneurysm dimensions identified in each imaging plane. (A) Right anterior oblique caudal view 46 × 40 mm; left anterior oblique caudal view 42 × 36 mm. (B) Right anterior oblique caudal view 39 × 32 mm; left anterior oblique caudal view 43 × 30 mm. (C) Right anterior oblique caudal view 30 × 22 mm; left anterior oblique caudal view 31 × 32 mm showing aneurysm is completely thrombosed.

Compared with initial imaging and 3D rendering of the aneurysm at baseline (Figure 4A), repeat CCTA 1 month later demonstrated complete occlusion of the septal branch and aneurysm by coils, with an interval reduction in aneurysm size (Figure 4B). At 6 months, the aneurysm measured 31 × 32 mm with complete obliteration of the aneurysmal cavity (Figure 4C). The patient remained asymptomatic, was physically active, and had experienced no interval arrhythmias or ischemic events.

## Potential Pitfalls

Coiling of CAAs carries notable risks, including incomplete occlusion, coil embolization, mechanical injuries such as vessel dissection or perforation, aneurysm rupture, and ischemia or myocardial infarction. Given the septal location of the aneurysm, there was also a risk for possible conduction disturbances.

## Conclusions

Percutaneous management of giant CAAs arising from septal perforating branches has not been previously described to our knowledge. Although the long-term clinical significance of giant CAA remains unclear, the potential complications arising from these structures can be life threatening. When surgical approaches may be unfavorable and anatomy permits, transcatheter closure of a giant CAA can be beneficial. We report successful coiling of a giant CAA arising from a septal perforator, with full obliteration of the aneurysmal cavity and a considerable reduction in size of the aneurysm on follow-up imaging. Multimodality imaging, particularly CCTA, played a crucial role in diagnosis, procedural planning, and postprocedural assessment. Follow-up imaging demonstrating depressurization of the aneurysm and obliteration of flow into the defect is essential in assessment of procedural success.

**Visual Summary:**
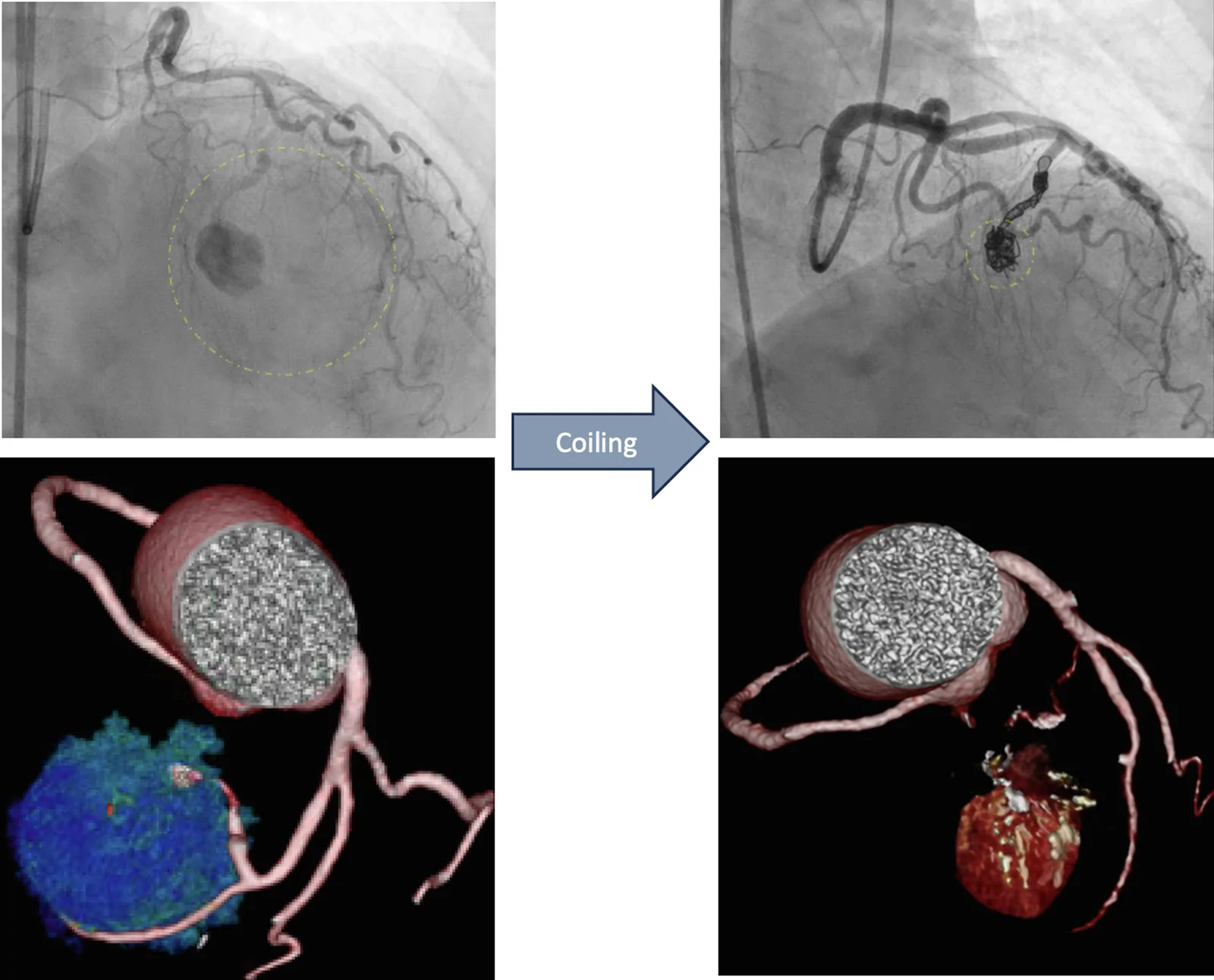
Successful Coiling of Giant Coronary Artery Aneurysm Arising From a Septal Perforator Branch

## Funding Support and Author Disclosures

Dr Mahoney has served as a consultant for Edwards Lifesciences, Medtronic, Abbott, and Boston Scientific. All other authors have reported that they have no relationships relevant to the contents of this paper to disclose.

**Take-Home Messages**

• When anatomy permits, transcatheter coiling via a feeding branch can fully obliterate a giant septal CAA and represents a viable alternative to surgical approaches.
• Multimodality imaging is essential for diagnosis, procedural planning, and postprocedural assessment.

## Appendix

Video 1 Cine Angiography of Coronary Aneurysm Coronary angiography in right anterior oblique (A) and anteroposterior (B) cranial views showing a large septal perforator branch giving rise to a small contrast-filled chamber surrounded by a large, presumably thrombosed and calcified mass.

Video 2 Engaging the Aneurysm Initial wiring of the aneurysm with repeated engagement of proximal septal branch. A longer BMW wire was subsequently advanced to successfully engage the aneurysm.

Video 3 Coiling of the Aneurysm A total of 4 coils were placed: 2 coils within the aneurysm to promote thrombosis, followed by 2 coils within the septal branch to completely occlude the flow to the aneurysm.
